# Electrochemical sensor based on iron-based metal-organic framework nanocomposite derived from acid mine drainage for the detection of lead (II) ions in water

**DOI:** 10.1007/s11356-026-37670-7

**Published:** 2026-03-25

**Authors:** Ntombenhle Maria Masanabo, Usisipho Feleni, Richard Moutloali, Lawrence Mzukisi Madikizela, Lueta-Ann de Kock

**Affiliations:** Institute for Nanotechnology and Water Sustainability, College of Science, Engineering and Technology, University of South Africa, Florida Science Campus, Roodepoort, 1710 South Africa

**Keywords:** Acid mine drainage valorization, Electrochemical sensor, Fe**-**alginate MOF, Fe-BTC, Lead detection, Metal-organic framework

## Abstract

Lead (Pb^2+^) in water poses significant health risks, which require sensitive and cost-effective detection techniques. This study addresses this challenge by presenting a novel electrochemical sensor based on Fe-alginate metal–organic framework (Fe-alg-MOF) composite. This composite was prepared by leveraging the high iron content in acid mine drainage (AMD) to form Fe-alg-MOF composite via an in situ synthesis process. Characterization revealed a semi-amorphous material with irregularly shaped Fe-MOF particles embedded within the alginate network, and a surface area of 302.4 m^2^/g. The Fe-alg-MOF was incorporated with polytetrafluoroethylene (PTFE) on a glassy carbon electrode (GCE) to fabricate a Fe-alg-MOF/PTFE/GCE sensor. Square wave voltammetry (SWV) was employed for Pb^2+^ detection at − 0.59 V, achieving a detection limit of 1.0 µM and a linear range of 4–50 µM. Furthermore, the practical applicability of the sensor was demonstrated by high recovery rates (~ 100%) in real wastewater effluent samples. Compared to conventional sensors, this platform utilizes an AMD waste derived material, demonstrating significant potential as a cost-effective and reliable tool for monitoring lead contamination in environmental waters.

**Supplementary Information:**

The online version contains supplementary material available at 10.1007/s11356-026-37670-7.

## Introduction

The global population and industrial activity have intensified concerns regarding lead (Pb^2^⁺) contamination in water. This contamination primarily results from mining, agriculture, plumbing, and waste disposal (Gmati et al. 2026; Xu et al. 2025; Zafar et al. 2025). Exposure to Pb^2^⁺ poses severe health threats-especially to children, leading to irreversible neurological damage and developmental delays (Bahiraei et al. 2026; Córdoba-Gamboa et al. 2023; Cüce et al. 2025; Schneider 2023). Consequently, numerous techniques have been developed for the detection of Pb^2+^ in environmental samples.

Conventional analytical detection methods for Pb^2+^ detection include chromatography, inductively coupled plasma spectroscopy, and flame atomic adsorption spectrometry (FAAS), among others (Arjomandi and Shirkhanloo 2019; Bulska and Ruszczyńska 2017; Ghorbani et al. 2018). Despite their high accuracy and sensitivity, these conventional methods require sample preparation, instrument training, and are expensive to run. As a result, alternative technologies for detecting Pb^2+^ in water are required.

Electrochemical sensors have emerged as efficient alternatives for detecting Pb^2+^ in water due to their rapid response, high sensitivity and cost-effectiveness (Xu et al. 2024). However, the sensing performance of a sensor is dependent on the electrode material (Ansari et al. 2025; Masanabo et al. 2023). Bare electrodes often suffer from poor selectivity, surface passivation loss, and high background noise (Nepfumbada et al. 2024). Thus, extensive research has focused on electrode modification using a range of electroactive materials to improve sensitivity, selectivity and overall sensor performance.

Metal–organic-frameworks (MOFs), particularly Fe-based MOFs have gained attention due to their high surface area, tuneable porosity, low toxicity and redox-active iron centres. Moreover, the presence of unsaturated iron coordination sites within Fe-MOFs, act as electroactive centers that promote strong analyte-MOF interactions, thereby enhancing electrochemical sensitivity (Hosseinzadeh & Rodriguez-Mendez 2023). For example, Hassani and co-workers exploited the high electrocatalytic activity of Fe-MOF based on BTC = benzene-1,3,5-tricarboxylic acid ligand for the detection of As^3+^ (Hassani et al. 2023). The Fe-BTC modified with gold nanoparticles exhibited a high electroactive surface area and rapid electron transfer, enabling efficient As^3+^ determination with a detection limit of 0.2 nM due to the strong interaction between Fe-MOF and the As^3+^ (Hassani et al. 2023). Electrochemical Pb^2^⁺ detection using Fe-based MOF platforms has also been widely reported. For instance, an immunosensor based on NH_2_-MIL-53(Fe) MOF was developed for the detection of Pb^2+^ (Singh et al. 2024). The incorporation of a monoclonal antibody afforded a highly sensitive sensor with a detection limit of 9.5 ppb and a wide linear range of 50–1000 ppb (Singh et al. 2024). While such antibody-assisted strategies provide excellent sensitivity, they inherently increase sensor complexity and cost. Beyond immunosensors, Fe-MOF based electrochemical platforms without biological recognition elements have also been explored. Zheng and co-workers developed a graphene@Fe-based MOF composite electrode for the simultaneous electrochemical detection of Cd^2+^ and Pb^2+^, achieving a low limit of detection of Pb^2+^ (Zheng et al. 2025). In this system, the Fe-MOF provided abundant electroactive sites that promoted strong interactions with Pb^2+^, thereby improving analyte accumulation at the electrode surface and sensitivity (Zheng et al. 2025). Beyond single-metal MOF systems, bimetallic hybrid sensors have also been explored for Pb^2+^ detection. Doan and co-workers reported on a bimetallic Fe,Mg-MOF/@reduced graphene oxide hybrid sensor system for Pb^2+^ detection (Doan et al. 2025). This bimetallic hybrid sensor achieved a detection limit of 0.01 µM, which was attributed to the high conductivity of the bimetallic Fe,Mg-MOF (Doan et al. 2025).

Despite Fe-MOF-based sensors demonstrating remarkable performance in Pb^2+^ sensing, most reported Fe-MOF-based sensors involve complex multi-component fabrication strategies, and high-purity chemical precursors, which increase material complexity and costs (Singh et al. 2024; Zheng et al. 2025). Moreover, strong acids and reducing agents are frequently employed during the preparation of these sensor systems (Doan et al. 2025). These challenges highlight the need for alternative Fe-MOF-based Pb^2+^ sensing platforms that combine material simplicity, cost-effectiveness, and sustainability. Efforts of promoting circular economy approaches have driven the use of waste materials as alternative precursors for MOF synthesis. Several studies (Dyosiba et al. 2016; Moumen et al. 2024; Teng et al. 2024) have demonstrated the feasibility of synthesizing MOFs from waste sources such as polyethylene terephthalate (PET) bottles and acid mine drainage (AMD). However, the synthesized MOF materials are predominantly used in adsorption applications rather than electrochemical sensing (Dyosiba et al. 2016; Moumen et al. 2024; Teng et al. 2024). The translation of waste-derived MOFs into functional sensing platforms therefore still remains underexplored. Furthermore, MOFs have a tendency to aggregate, and their poor mechanical stability can compromise electrode integrity and long-term performance, hindering practical MOF-sensor deployment (Chakraborty et al. 2024; Molavi et al. 2024; Shah et al., 2019).

Polymer-MOF composites have emerged as an effective approach to overcome the poor mechanical stability and aggregation of pristine MOFs while retaining their electrochemical activity. Alginate, a non-toxic, biodegradable polysaccharide polymer can serve as a structural template during in situ alginate-Fe-MOF composite formation, preventing framework collapse and ensuring uniform (Chai et al. 2022; Moumen and El Hankari 2024; Zhang et al. 2024). This in situ incorporation of alginate-MOF approach can subsequently improve electrode stability and electrochemical performance. Alginate-MOF composites have been explored for sensing biomolecules such as β-lactamase β-lactamase (Lian and Yan 2019) and thrombin (Lin et al. 2020). However, their application in heavy-metal detection remains unreported. Herein, this study reports for the first time, a Fe-alginate-MOF-based electrochemical sensor for the detection of Pb^2^⁺ in water. The composite was synthesized via an in situ approach, in which alginate was combined with an iron-rich synthetic acid mine drainage (AMD) and subsequently coordinated with trimesic acid to form Fe-alginate-MOF. This approach integrated the electroactive properties of the Fe-MOF with alginate as a template while simultaneously valorising AMD as a secondary iron source. The integration of the electroactive properties of Fe-based MOFs within alginate provides an efficient and sustainable platform for Pb^2^⁺ sensing, with enhanced electron transfer and selectivity.

## Experimental

### Chemicals and reagents

The chemicals used in this study were: sodium alginate_,_ (99%), iron (III) chloride hexahydrate (97%), aluminum nitrate nonahydrate (99%), magnesium sulfate heptahydrate (≥ 98%), manganese sulfate hydrate (≥ 99.99%), hydrochloric acid (30%), sulfuric acid (98%), trimesic acid **(**95%), disodium hydrogen phosphate dibasic (≥ 99.5%), sodium phosphate monobasic dihydrate (≥ 99%), potassium chloride (98%), sodium hydroxide (99%), polytetrafluoroethylene (60 wt.% DISPER) solution, potassium hexacyanoferrate (III) (99%), potassium hexacyanoferrate (II) (99%), sodium acetate monohydrate (99%), lead nitrate (≥ 99.0%), glacial acetic acid (≥ 99%), and ethanol (98%). All of these chemicals were purchased from Merck (Johannesburg, South Africa) and used without further purification.

### Instrumentation

Samples prepared in this study were characterized with X-ray diffraction (XRD), scanning electron microscopy (SEM) coupled with an energy dispersive X-ray spectroscopy (EDS) detector, Fourier transform infrared spectroscopy (FTIR), Raman spectroscopy, Transmission Electron Microscopy (TEM) and Brunauer–Emmett–Teller (BET). XRD analysis was performed with a Rigaku SmartLab X-ray diffractometer (Rigaku, Tokyo, Japan) equipped with a Cu K $\alpha$ X-ray source of wavelength 0.15418 nm. The measurements were conducted at a voltage and current of 45 kV and 200 mA, respectively. Surface morphology and elemental composition of the prepared samples were examined using SEM (JSM-IT300 Joel, Tokyo, Japan) coupled with EDS (Oxford instruments, Abingdon, UK). Prior to SEM–EDS analysis, samples were affixed to double-sided carbon tape on a sample holder disc, sprayed with canned air to remove loose particles, and then coated with gold using a rotary-pumped spatter coater (Q150R ES, Quorum Technologies, UK). Afterwards, an electron beam accelerated at 20 kV was used to probe the samples in the SEM instrument chamber. TEM imaging was obtained using a JEM 2100 TEM (JEOL JEM, Tokyo, Japan) operating at 200 kV, Prior to TEM analysis, the samples were sonicated in ethanol for 30 min and then dispersed onto a carbon-coated copper grid for examination. FT-IR spectroscopy (Perkin Elmer spectrum 100 FT-IR, Massachusetts, USA) was used to determine different functional groups using the KBr pellet method. Raman spectroscopy results were obtained using the WITec alpha300 Series Raman spectrometer (Oxford instruments, Abingdon, UK) with a laser power wavelength of 532 nm.

Electrochemical experiments were recorded using a portable PalmSens4 (PalmSens Compact Electrochemical Interfaces, Houten, The Netherlands) computer-controlled potentiostat with a standard three-electrode system. A bare glassy carbon electrode (GCE) with diameter of 3 mm and modified GCE, respectively served as a working electrode, a platinum wire was used as a counter-electrode, and an Ag/AgCl electrode was utilized as a reference electrode. Electrochemical properties of the modified electrodes were investigated using cyclic voltammetry (CV) and electrochemical impedance spectroscopy (EIS) techniques.

### Synthesis of Fe-alginate-MOF composite using synthetic acid mine drainage

The synthesis of Fe-MOF-alginate composite involved two sequential stages: (i) preparation of synthetic acid mine drainage, followed by (ii) in situ synthesis of the target composite material. This approach ensured controlled integration of iron-based metal–organic framework (Fe-MOF) constituents within the alginate matrix.

#### Preparation of synthetic acid mine drainage

At the time of the experiments, access to real AMD waste was limited due to logistical constraints; therefore, synthetic AMD was used. In addition, synthetic AMD made it possible to have controlled and reproducible experimental conditions as well as providing systematic evaluations of the materials’ performance. The synthetic sAMD was prepared based on reported concentrations from a number of publications (Alegbe et al. 2019; Bell et al. 2001; Kefeni and Mamba 2021; Kefeni et al. 2017; Masindi 2017; Mwewa et al. 2019; Mxinwa et al. 2020; Novhe et al. 2016).

A mixture of metal salts listed in Table 1 was dissolved in 1 L of deionized water to form synthetic AMD. The pH of the prepared synthetic AMD was measured to be 2.08.

**Table 1:** Composition of synthetic acid mine drainage

Metal salt	Metal ion concentration (ppm)	Counter ion concentration (ppm)
FeCl_3_·6H_2_O,	2847	5420
Al(NO_3_)_3_·9H_2_O	232	160
MgSO_4_·7H_2_O	312	1235
MnSO_4_·H_2_O	65	114

#### In situ synthesis of Fe-alginate-MOF composite using synthetic acid mine drainage

**Scheme 1:**
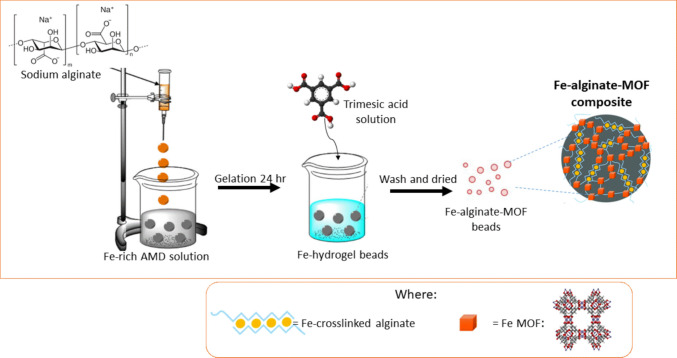
Schematic representation of Fe-alginate-MOF beads synthesis

The first step of the procedure was to prepare a 2% (w/v) alginate solution, and to transfer the prepared alginate solution into a burette. Then, 100 mL of alginate solution was added dropwise to 200 mL of sAMD solution at room temperature to form Fe-alginate (Fe-alg) hydrogel beads. The beads were then allowed to gelate for 24 h to ensure complete gelation. After 24 h, the Fe-alg hydrogel beads were filtered and washed with deionized water to remove any un-crosslinked metal ions. The purified Fe-alg hydrogel beads were then immersed in a solution containing 30 mg/mL of trimesic acid in 100 mL ethanol for 24 h to generate Fe-alginate-MOF (Fe-alg-MOF) composite as shown in Scheme 1. The resulting composite was washed with deionized water, followed by ethanol to remove any remaining trimesic acid before being air-dried.

### Synthesis of Fe-MOF

The synthesis of iron-based metal–organic framework (commonly known as Fe-BTC) was conducted following established procedures with some modifications (Steenhaut et al. 2020). Fe-MOF was synthesized by preparing two solutions. One solution was composed of 1.42 g trimesic acid and 0.80 g NaOH, dissolved in 125 mL of deionized water. The other solution contained 2.25 g iron (III) chloride hexahydrate dissolved in 125 mL of deionized water. The NaOH/trimesic solution was added dropwise to the iron solution under continuous stirring for 24 h to yield an orange Fe-MOF powder. The orange powder was recovered from the solution by centrifugation and subsequently stored in an airtight container. Fe-MOF was used as a control and for comparative studies.

### Preparation of a Fe-alginate-MOF-modified glassy carbon electrode

Prior to surface modification, the GCE was polished with a Buehler-felt polishing pad in alumina slurries of 1.0, 0.3 and 0.05 µm, respectively. Thereafter, the polished electrode was cleaned in deionized water and ethanol for 10 min, to remove residual alumina. A Fe-alg-MOF suspension was prepared by mixing 1 mL of ethanol with 1 g of finely crushed Fe-alg-MOF beads. A total of 8 µL of the Fe-alg-MOF suspension was drop-casted on the GCE and air-dried to form a Fe-alg-MOF/GCE. For comparison, bare GCE, Fe-alg/GCE, and Fe-MOF/GCE were also prepared following the same procedure.

### Preparation of a Fe-alginate-MOF/polytetrafluoroethylene -modified glassy carbon electrode

In preparation for surface modification, a bare GCE was firstly abraded with a Buehler-felt polishing pad. Thereafter, it was polished with 1.0 µm, 0.3 µm, and 0.05 µm alumina slurries, and sonicated consecutively for 10 min in ethanol and deionized water. The polished GCE was modified by drop-casting 8 µL of the Fe-Alg-MOF –ethanol suspension onto the GCE surface, followed by the application of 2 µL of polytetrafluoroethylene (PTFE) solution (0.05 wt%). The modified GCE was air-dried prior to electrochemical studies. For comparison, bare GCE, Fe-alg/PTFE, and Fe-MOF/PTFE-modified electrodes were also prepared.

### Real wastewater samples

The practical application of the developed sensor was established by determining the concentration of Pb^2+^ in wastewater samples collected from Zwelitsha wastewater treatment plant, Eastern Cape, South Africa. The collected wastewater samples constituted of both influent and effluent. The samples were collected in 1-L glass bottles using Grabs method. The samples were purified by filtering through 11-µm Whatman filter paper to eliminate suspended solid materials, and the pH of wastewater samples was adjusted to the optimal pH, before electrochemical analysis.

### Electrochemical experiments

Electrochemical sensing experiments were performed using CV, differential pulse voltammetry (DPV) and square wave voltammetry (SWV) techniques in 1.0 M electrolyte solution. Nitrogen gas was used to degas the electrochemical cells for approximately 10–20 min before performing any electrochemical experiments. EIS investigation was carried out using 1 mM [Fe(CN)6]^3–/4–^ prepared in 0.1 M KCl, while the redox properties of the modified electrode were determined using 0.1 M acetate-based electrolyte solution (ABS) under nitrogen. The ABS solution was prepared by adding appropriate amounts of sodium acetate monohydrate and glacial acetic acid to form 0.1 M and 1.0 M ABS.

CV experiments were carried out by applying a wide potential range of − 1.0 V to + 1.0 V. DPV and SWV responses of Pb^2+^ detection were recorded between − 0.9 V and − 0.3 V, at an E-step 0.01 V, E-pulse 0.2 V and t-pulse 0.05 s. Aqueous solutions of Pb^2+^ (0.1 to 1.0 mM) were prepared from lead nitrate. All detection experiments were performed at room temperature.

## Results and discussion

### Characterisation of Fe-alginate-MOF composite

**Fig. 1:**
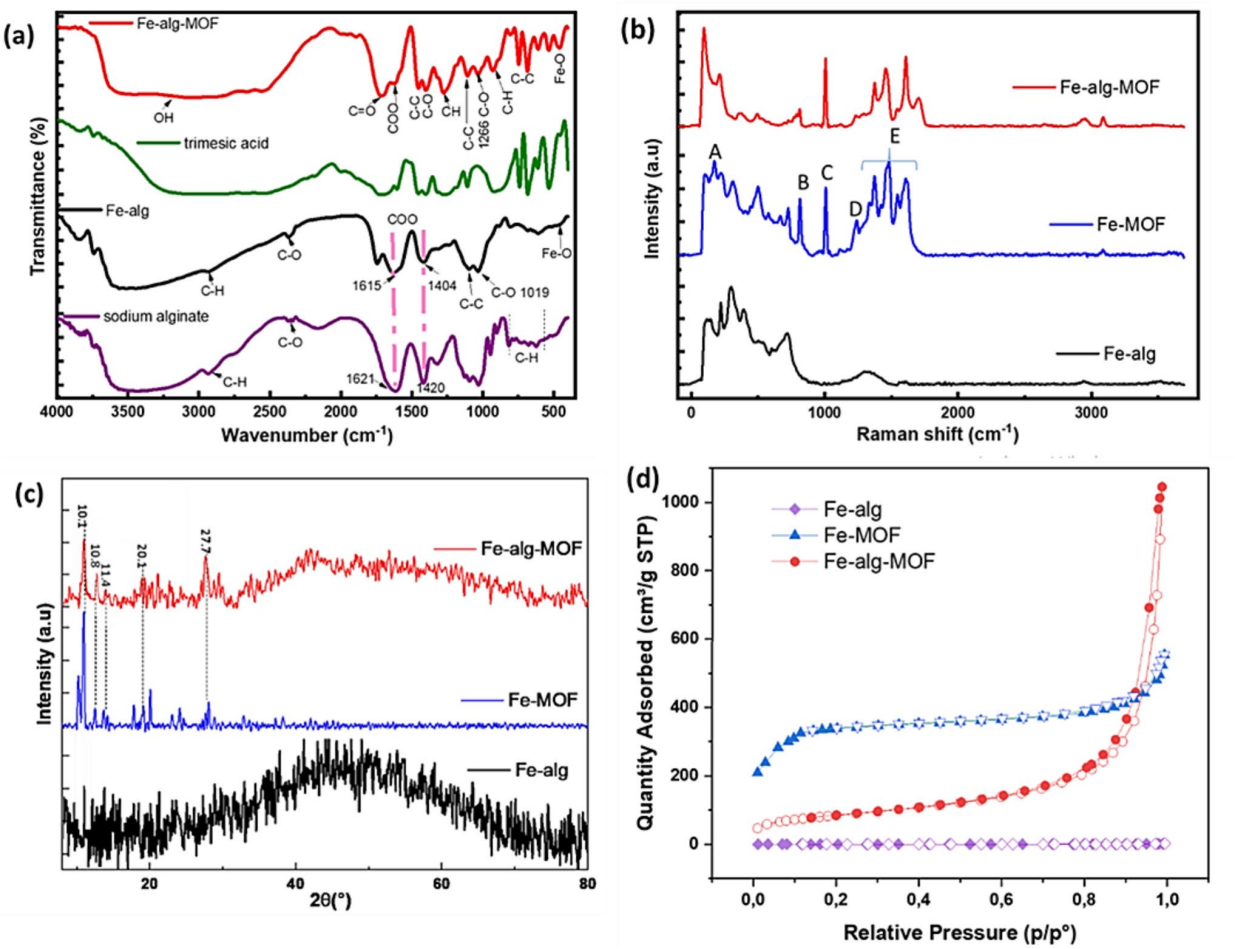
FTIR spectrum of (**a**) different precursors and synthesized materials, (**b**) Raman spectrum of different synthesized material and (**c**) XRD spectrum of synthesized materials and (**d**) N_2_ adsorption–desorption isotherms at 77 K (The filled markers represent adsorption isotherms and the open markers refer to the desorption isotherms)

The addition of sodium alginate to the Fe-rich synthetic AMD resulted in Fe-cross-linked alginate beads denoted as Fe-alg. As a result of crosslinking sodium alginate with Fe^3+^, the symmetrical and asymmetrical vibrations of COO- group exhibited a red shift as observed in Fig. 1a. These shifts in the asymmetrical and symmetrical COO^−^ in Fe-alg confirmed the complexation of metal ion and carboxylate group (Badita et al. 2020; Bierhalz et al. 2014). Interestingly, there was an extended peak around ~ 1700 cm^−1^ for Fe-alg, possibly originating from Fe^3+^ also co-ordinating with the β-D-mannuronic (M) and α-L-guluronic (G) blocks. The Fe-alg spectrum also had a broad band around 3500 cm^−1^ which was attributed to OH group vibration, and a band around 2400 cm^−1^ due to the frequency-doubled peak of C-O stretching as observed by Li et al. (2021). Other less prominent peaks between 1100 and 1000 cm^−1^ were assigned to C–O and C–C stretching within alginate. The peaks below 800 cm^−1^ were related to the C-H bending of guluronic acid and mannuronic acid from alginate. A peak below 600 cm^−1^ indicated that there was co-ordination bonding between Fe^3+^ and alginate polymer (Fig. 1a), similar to findings reported by Hernández et al. (2010).

The Fe-alg was treated with trimesic acid to form Fe-alginate-MOF (Fe-alg-MOF) composite in Fig. 1a. The composite was dominated by the alginate matrix, hence the absence of the strong bands associated with MOF formation normally seen at 1600 cm^−1^ and 1400 cm^−1^ region (Alvares et al. 2023; Le et al. 2022). However, bands corresponding to the symmetrical alginate COO- groups showed a red shift to 1600 cm^−1^ region, indicating complexation upon the addition of trimesic acid. Moreover, a peak at 1718 cm⁻^1^ attributed to the C = O bond from trimesic acid was observed within the composite, confirming the integration of trimesic acid within alginate. The Fe-alg-MOF composite FTIR spectrum, also displayed peaks at 1461 cm^−1^ and 1412 cm^−1^ attributed to C–C stretching within the benzene ring and C-O stretching of trimesic acid, respectively. The spectral region between 1300 and 900 cm^−1^ displayed signals representing both in-plane and out-of-plane bending vibrations of C-H bonds from the benzene ring. Furthermore, characteristic peaks in the range of 700–500 cm^−1^ were identified as bending vibrations of C–C bonds from the benzene ring. and strong bands around 800–600 cm^−1^ attributed from the CC stretching of trimesic acid. All the observed spectral peaks associated with Fe-alg-MOF were similar to the peaks reported by Li et al. (2021), Mahalakshmi and Balachandran (2014), and Zhou et al. (2022). Despite repeated ethanol washing of the composite, the FTIR spectrum of Fe-alg-MOF retained characteristic peaks associated with uncoordinated trimesic acid. Therefore, additional characterization techniques had to be employed to confirm the formation of Fe-alg-MOF.

Raman spectroscopy was further used to characterize the synthesized materials and analyze their molecular vibrational modes as shown in Fig. 1b. The Raman spectrum of the synthesized Fe-MOF revealed Fe ions integrated into the lattice structure (Region A). The spectrum further displayed peaks associated with the oscillation of the trimesic acid benzene ring at 832.5 cm⁻1 and 1026.9 cm⁻1 (Regions B and C). Additionally, the bonding of C-O-Fe in region D confirmed the bonding of iron trimer at 1234.2 cm^−1^, while the presence of water molecules within the MOF framework was observed at region E (1409–1611 cm^−1^). All the observed Raman shifts of the synthesized Fe-MOF were similar to that of the commercialized Fe-BTC MOF (Le et al. 2022; Li et al. 2017; Nivetha et al. 2020), indicative of a pure Fe-MOF. Furthermore, the Raman spectra of Fe-alg-MOF composite exhibited shifts related to Fe-MOF, with additional peaks from the alginate backbone vibrations below 1000 cm⁻1 and carboxylate stretching vibrations above 1000 cm⁻1 (Hernández et al. 2010).

Additionally, the XRD spectra in Fig. 1c, showed that Fe-alg was amorphous in nature which is typical for a polymer. However, Fe-alg-MOF composite was semi-amorphous with diffraction peaks at 10.1°, 10.8°, 20.1° and 27.7 ^o^ associated with Fe-BTC MOF (Devi et al. 2020; Huo and Yan 2012; Sun et al. 2022) being present in the composite. The absence of other foreign peaks within the composite indicated a pure Fe-alg-MOF. The surface area, pore size and pore volume of the synthesized materials were determined using BET. The results of the BET isotherms are presented in Fig. 1d, with corresponding parameters summarized in Table 2. Fe-alg exhibited a Type II isotherm, characteristic of a macroporous material with a relatively small surface area of 0.4 m^2^/g. In contrast, Fe-MOF had an exceptionally high surface area of 1297.3 m^2^/g, with a Type IV isotherm indicating micropore adsorption at low pressure and mesopore adsorption at higher pressures. In addition, the in situ synthesized Fe-alg-MOF, had a higher surface area of 302.4 m2/g, compared to Fe-alg. The high surface area of Fe-alg-MOF further indicated the successful formation of Fe-MOF within the Fe-alg matrix, Furthermore, Fe-alg-MOF isotherm represented a Type IV isotherm with larger mesopores compared to Fe-alg. This was indicative of a mesoporous material with enhanced pore channels and surface accessibility possibly due to the incorporation of Fe-MOF (Ambroz et al. 2018; Rahman et al. 2021).

**Table 2:** BET data of synthesized materials

	BET surface area (m^2^/g)	Pore size (Å)	Pore volume (cm^3^/g)
Fe-alg	0.4	116.0	0.004
Fe-MOF	1 297.3	21.3	0.4
Fe-alg-MOF	302.4	98.6	1.6

**Fig. 2:**
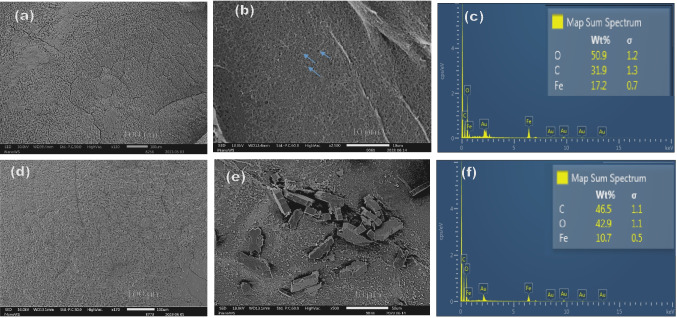
Different magnified SEM images of (**a**) surface morphology of Fe-alg; (**b**) cross-section of Fe-alg, (**c**) EDX of Fe-alg, (**d**) surface morphology of Fe-alg-MOF, (**e**) cross-section of Fe-alg-MOF and (**f**) EDX of Fe-alg-MOF

**Fig. 3:**
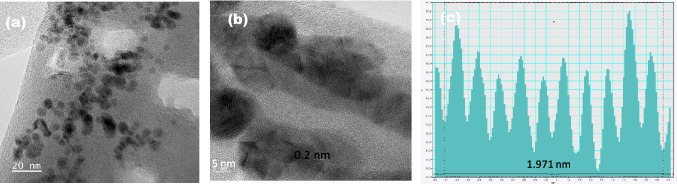
Different magnified TEM images at (**a**) 50 nm, (**b**) 5 nm with particle size of 0.2 nm ± 0.5 and (**c**) Size distribution of Fe-MOF particles within alginate

The surface morphology was also studied and from the data obtained in Fig. 2. Fe-alg (Fig. 2a) had wrinkled morphology and the cross section of Fe-alg (Fig. 2b) showed the absence of nanoparticles within the alginate matrix. The elemental analysis of Fe-alg only showed the presence of oxygen, carbon, and iron as depicted in Fig. 2c. Similarly, Fe-alg-MOF (Fig. 2d) had similar wrinkled morphology as Fe-alg. However, the SEM cross-section of Fe-alg-MOF (Fig. 2e) revealed irregularly shaped particles embedded within the alginate matrix. Additionally, the elemental analysis of Fe-alg-MOF showed the presence of oxygen, carbon, and iron and an increase in carbon and oxygen content from Fe-alg to Fe-alg-MOF due to the addition of trimesic acid. TEM imaging further confirmed the presence of irregularly shaped, agglomerated particles within the Fe-alg-MOF composite as seen in Fig. 3a–c.

### Electrochemical behavior of Fe-alginate-MOF composite

**Fig. 4:**
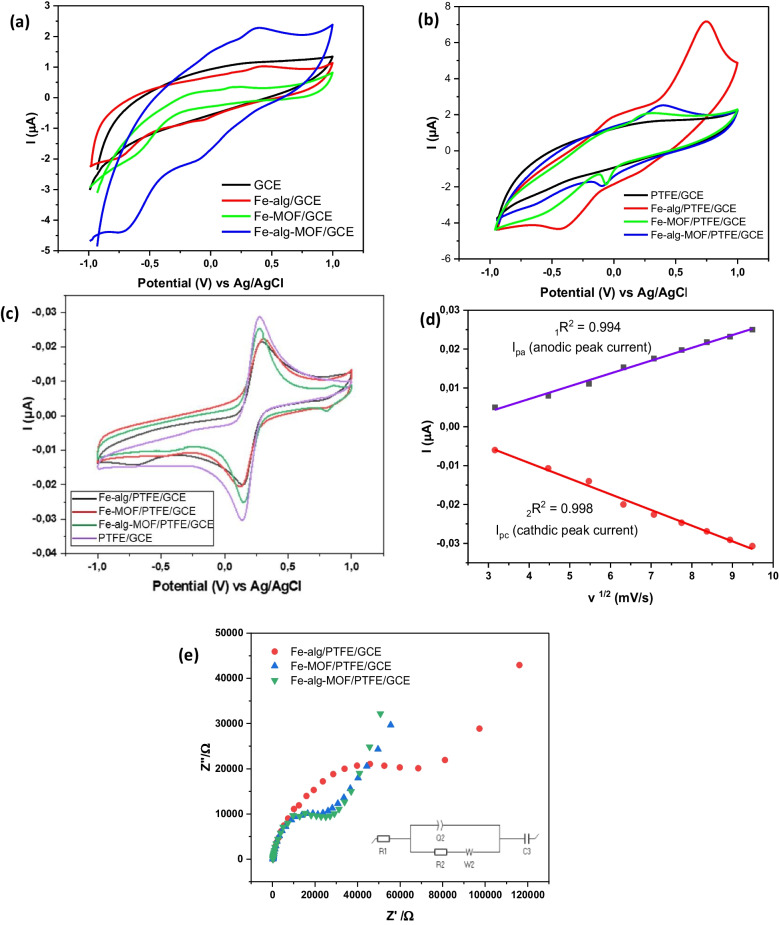
CV responses of different modified GCEs (**a**) without a binder (**b**) with a binder in 0.1 M ABS without the presence of analyte. (**c**) CV responses in 1 mM [Fe(CN)₆]^3^⁻/^4^⁻ solution at scan rate of 10 mV/s (**d**) Graph of current vs. square root of scan rate, (**e**) Nyquist plots of synthesized materials in 1 mM [Fe(CN)₆]^3^⁻/^4^⁻ solution

Cyclic voltammetric measurements were employed to study the redox nature of bare GCE, modified Fe-alg, Fe-MOF, and Fe-alg-MOF modified electrodes, respectively, under 0.1 M acetate electrolyte solution (Fig. 4a). CV analysis of bare GCE showed no redox signal, indicating a clean GCE surface. However, after modifying the bare GCE with the respective materials, different redox peaks appeared indicating the presence of an iron active center within the materials. For instance, Fe-MOF showed one oxidation peak at 0.257 V and a reduction peak at − 0.606 V which were attributed to the Fe^3+^/Fe^2+^ redox couples (Zhang et al. 2020). In contrast, Fe-alg/GCE and Fe-alg-MOF/GCE exhibited one oxidation and two reduction peaks. The Fe-alg/GCE demonstrated an oxidation peak at 0.334 V and two reductions at − 0.582 V and − 0.048 V. The reduction at −0.582 V, was from Fe^3+/^Fe^2+^ while the peak at − 0.048 V was possibly due to the dissolution of metal ion within the alginate matrix. The redox peaks of Fe-alg-MOF/GCE shifted to the negative potential compared to Fe-alg/GCE, with an oxidation peak at 0.349 V and two reduction peaks at − 0.078 V and − 0.668 V, possibly because of the presence of Fe-MOF. In addition, Fe-alg-MOF-modified GCE showed a greater increase in current than Fe-alg/GCE and Fe-MOF/GCE. This could be due to the enhanced surface area of Fe-alg-MOF.

Further electrochemical studies had to be carried out in the presence of a binder because the synthesized materials would leach off the electrode surface during CV scans. Therefore, PTFE was chosen as a binder because of its availability and stability, serving as an inert binder without electrochemical activity as shown in Fig. 4b. The presence of a binder however resulted in a current decrease as seen in Fig. 4a and b. This reduction in current was possibly due to PTFE blocking some Fe-alg-MOF active sites. The redox profile of Fe-alg-MOF/PTFE modified electrode was analyzed in Fig. 4b and showed an oxidation peak at + 0.360 V, attributed to the Fe^2^⁺/Fe^3^⁺ redox couple, and a reduction peak at − 0.058 V, corresponding to the dissolution process involving the Fe(s)/Fe^2^⁺ couple. In contrast, the Fe-alg/PTFE modified GCE displayed two oxidation peaks: one at + 0.082 V, assigned to the re-oxidation of Fe^2^⁺ to Fe^3^⁺ (Feleni et al. 2020), and another at + 0.737 V, also related to the Fe^3^⁺/Fe^2^⁺ redox transition (Sundramoorthy et al. 2016). Additionally, a reduction peak at − 0.393 V, corresponding to the Fe^3^⁺/Fe^2^⁺ couple, was observed. The Fe-MOF/PTFE modified electrode (Fig. 4b) displayed a single oxidation peak at + 0.271 V, attributed to the Fe^3^⁺ to Fe^2^⁺ oxidation, along with a reduction peak at − 0.063 V, likely resulting from the reduction of Fe^2^⁺ to metallic iron (Fe⁰), possibly associated with metal dissolution due to iron hydrolysis (B. Zhang et al. 2020). The different redox peaks of the modified electrodes (GCE/PTFE/Fe-alg; GCE/PTFE/Fe-MOF and GCE/PTFE/Fe-alg-MOF) in the presence of PTFE were summarized in Table 3.

**Table 3:** Redox signals of synthesized material with a binder in the absence of analyte

Synthesized materials	Oxidation (V)		Reduction (V)
Fe-alg/PTFE	0.082	0.737	− 0.393
Fe-MOF/PTFE	0.271	-	− 0.063
Fe-alg-MOF/PTFE	0.360	-	− 0.058

The reproducibility and robustness of Fe-alg-MOF/PTFE/GCE was also evaluated using CV. The peak current recorded from three independently prepared electrodes showed a relative standard deviation (RSD) of 0.4%, indicating good reproducibility. In addition, the electrode retained 89 ± 4.5% of its initial current after 10 consecutive CV cycles, confirming acceptable electrochemical stability.

Peak-to-peak current separation (∆E_p_) and electroactive surface area (A) of the modified electrodes i.e., (GCE/PTFE/Fe-alg; GCE/PTFE/Fe-MOF and GCE/PTFE/Fe-alg-MOF) were measured using CV technique in 1 mM [Fe(CN)_6_]^3–/4–^ solution.

The peak-to-peak current separation was calculated as shown in Eq. (1):

1 $$\Delta {E}_{P}={E}_{pa}-{E}_{pc}$$

where ${E}_{pa}$ is the anodic peak potential and ${E}_{pc}$ is the cathodic peak potential (Mkhohlakali, 2020; Ndebele and Nyokong 2023).

From Fig. 4c, the $\Delta {E}_{P}$ value increased in the following order: bare GCE/PTFE (136 mV) < GCE/PTFE/Fe-alg-MOF (140 mV) < GCE/PTFE/Fe-alg (150 mV) < GCE/PTFE/Fe-MOF (175 mV). The increase in $\Delta {E}_{P}$ value after modification usually occurs because the electrode surface would be blocked by the electrocatalyst (Ndebele & Nyokong 2023). The $\Delta {E}_{P}$ data further suggests that Fe-alg-MOF had better electron transfer capabilities in Fe(CN)_6_^3−/4−.^ solution compared to Fe-alg and Fe-MOF with high $\Delta {E}_{P}$ values.

Figure 4d was employed to assess the electrochemical characteristics of the electrocatalyst in the [Fe(CN)_6_] ^3−/4−.^ solution. A linear relationship between the peak current and root of scan rate was obtained (Fig. 4d), which could suggest a reversible system. However, the peak-to-peak separation ($\Delta {E}_{P}$), exceeded 59 mV, which indicated Fe-alg/PTFE/GCE, Fe-MOF/PTFE/GCE and Fe-alg-MOF/PTFE/GCE were quasi-reversible (Trachioti et al. 2023). The quasi-reversible reaction was further evaluated to determine whether the quasi-reversible was due to slow kinetics or solution uncompensated resistance. A graph showing the relationship between the values of peak separation and square root of the scan rate at different scan rates was plotted as Fig. S1. A linear relationship was established, which was compatible with a quasi-reversible system that is due to the kinetic effect, since the uncompensated resistance was sufficiently small (Muhammad et al. 2016).

Since the prepared materials exhibited a quasi-reversible behavior, the effective electroactive surface area was calculated using the Eq. (2) (Muhammad et al. 2016):

2 $${I}_{p}=\left(2.99\times {10}^{5}\right)nAC({\alpha nDv)}^{1/2}$$

where I_p_ is a peak current (in ampere, A), υ is the rate at which potential is scanned (scan rate in Vs^−1^), A is the electroactive area of the electrode, D is the diffusion coefficient (7.6 × 10^− 6^ cm^2^ s^−1^) of ferrocyanide solution (Ndebele and Nyokong 2023; Zhou et al. 2023), n is the number of electrons transferred in the electrochemical reaction (*n* ≈ 1), C is the concentration in mol cm^−3^ of the reactant species of [Fe(CN) _6_] ^3−/4−^, α is the charge transfer coefficient which is ~ 0.5 in an irreversible or quasi-reversible system (Muhammad et al. 2016).

The effective surface area of PTFE/GCE, Fe-alg/PTFE/GCE, Fe-MOF/PTFE/GCE, and Fe-alg-MOF/PTFE/GCE were determined to be 0.263 cm^2^, 0.127 cm^2^, 0.143 cm^2^, and 0.155 cm^2^, respectively. These results further support the data obtained from the $\Delta {E}_{P}$ values whereby Fe-alg-MOF has a more effective electroactive surface area compared to Fe-alg and Fe-MOF.

Electrochemical impedance spectroscopy (EIS) experiments were conducted using 1 mM [Fe(CN)_6_]^3–/4–^ solution (containing 0.1 M KCl) to determine the electron transfer resistance (R_ct_), as well as to compare the electron transfer characteristics of Fe-alg/PTFE/GCE, Fe-MOF/PTFE/GCE, and Fe-alg-MOF/PTFE/GCE. In a Nyquist plot, the high frequency region indicates the process at which the electrode surface is controlled by kinetics, and the low frequency represents the diffusion process. The diameter of the semi-circular portion in the lower frequency is equivalent to the electron transfer resistance (Rct) (Idris et al. 2022). According to the Nyquist diagram (Fig. 4e), the Fe-alg presented the largest R_ct_, of 78,400 Ω, whereas Fe-MOF and Fe-alg-MOF showed lower R_ct_ of 34,722 Ω and 31,363 Ω, respectively. The Fe-alg-MOF showed the lowest R_ct_, of 31,363 Ω, indicating that the composite can promote electron transport on the electrode surface compared to the Fe-alg (Fig. 4e). The impedance data were fitted using a modified Randles equivalent circuit composed of the solution resistance (R_1_), a constant phase element (Q_2_), charge transfer resistance (*R*^2^), Warburg impedance (W_2_), and a capacitive element (C_3_). The experimental results and fitted data showed excellent agreement (low χ^2^), confirming the suitability of the selected equivalent circuit.

The observed redox shifts in the CV curve (Fig. 4b) can be attributed to the structural features of the Fe-alg-MOF composite and are consistent with the EIS results. The presence of Fe^3+^ center within Fe-alg-MOF provides multiple redox-active sites and enhanced electron-transfer pathways. Furthermore, the EIS results of Fe-alg-MOF show reduced charge-transfer resistances compared to the Fe-alg and Fe-MOF, indicative of an improved electron transport and redox-active sites. These findings suggest that the synergetic interaction between Fe centers and alginate can provide redox-active sites and improve conductivity influencing the redox behavior seen in Fig. 4b.

### Electrochemical behavior of Fe-alginate-MOF composite in the presence of analyte

**Fig. 5:**
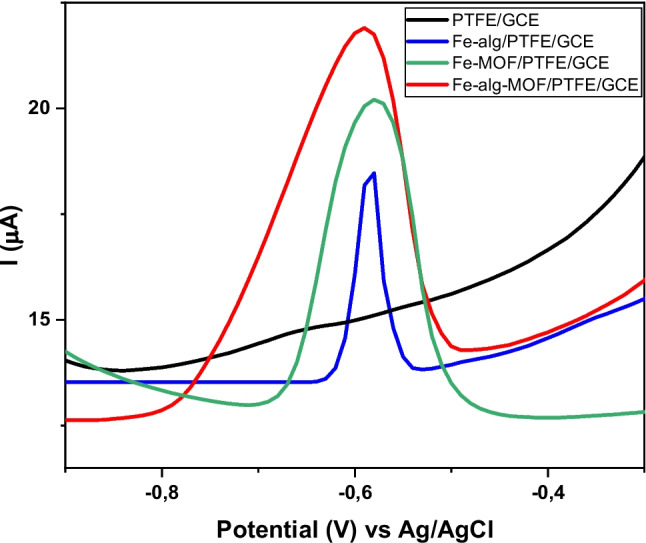
DPV of different modified electrodes in the presence of 1 mmol/L Pb^2+^, in 0.1 M ABS

The performance of the modified electrodes for Pb^2+^ detection was evaluated using DPV as depicted in Fig. 5 as it is more sensitive compared to CV**.** Based on the results, there was no detectable signal for Pb^2^⁺ when using the unmodified PTFE/GCE. The Fe-alg/PTFE- and Fe-MOF/PTFE-modified electrode displayed a peak at − 0.575 V, whereas Fe-alg-MOF/PTFE-modified showed a peak at − 0.593 V. These observed peaks were associated with the reduction of Pb^2+^ to Pb(s) as reported by numerous literatures (Bodkhe et al. 2020; Dahaghin et al. 2020; Poudel et al. 2022; Tran et al. 2023). Furthermore, the Fe-alg-MOF/PTFE-modified electrode displayed an enhanced current compared to Fe-alg and Fe-MOF modified electrodes, possibly, due to its better electroactive surface area and electron transfer kinetics. Therefore, Fe-alg-MOF/PTFE was further used for optimization.

### Electrochemical sensor for Pb^2+ ^with Fe-alginate-MOF composite

#### Effect of supporting electrolyte system

**Fig. 6:**
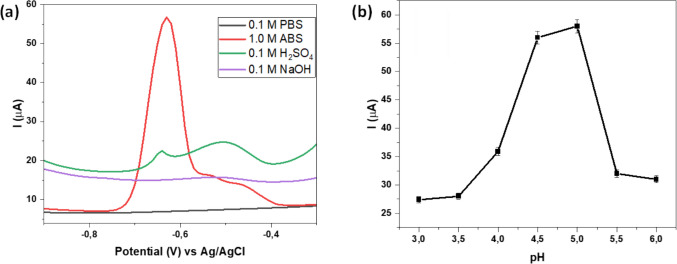
DPV response of the modified (**a**) Fe-alg-MOF/PTFE/GCE in various electrolyte solutions with 1 mmol/L Pb2⁺ and (**b**) Peak plot of current (µA) vs pH

As shown in Fig. 6a, the performance of Fe-alg-MOF/PTFE/GCE for Pb^2+^ detection in aqueous solutions was evaluated using various supporting electrolyte systems. Notably, no detectable signal Pb^2+^ signal was observed when 0.1 M NaOH or 0.1 M phosphate buffer solution (PBS) was used as supporting electrolyte. Instead, a response signal for the reduction of Pb^2+^ was observed when the modified electrode was subjected to acidic electrolyte solutions, such as ABS and sulfuric acid. This observation was attributed to the enhanced redox activity of Fe3⁺/Fe2⁺ in Fe-MOF under an acidic environment, which ensures that the Fe center of Fe-alg-MOF remains catalytically active when compared to the basic environment (Babu et al. 2010). Figure 6a further showed that a better signal for Pb^2+^ was obtained in ABS solution compared to sulfuric acid. However, the counter electrode exhibited electrode fouling following the experimental run when a 0.1 M ABS solution was utilized. This fouling was possibly initiated by the lower ionic strength of the buffer, reduced mass transport, and increased accumulation of reaction by-products (Hanssen et al. 2016; March et al. 2015; Zhao et al. 2022). Therefore, to limit the possible side reaction that occurred on the counter electrode, the concentration of ABS was increased to 1.0 M. This concentration increase resulted in a well-defined signal (Fig. 6a) with no side reactions observed on the counter electrode. Thus, 1.0 M ABS was selected as the optimum electrolyte solution with the Pb^2+^ ion reduction peak at 0.665 V.

#### Effect of pH

The selection of an appropriate pH for the electrolyte is essential to ensure that the electrochemical environment has optimal redox potential for the analyte, consequently improving precision and reliability. In this study, a pH range of 3–6 was investigated for the detection of Pb^2+^ using the Fe-alg-MOF/PTFE/GCE modified electrode (Fig. 6b). In this case, the influence of pH values beyond pH 6 was not conducted due to the potential precipitation of Pb^2+^ to Pb(OH)_2_, and weakened the electrolyte solution (Bodkhe et al. 2020; Dahaghin et al. 2020; Gumpu et al. 2017; Poudel et al. 2022). Figure 6b shows that the current increased with pH, reaching its maximum at pH 5 and decreasing after pH 5. These observations were because at low pH values, there exists a competition between H⁺ and Pb^2^⁺ ions for access to the active sites on the modified electrode surface (Cozmuta et al. 2012; Shakeri et al. 2012). Therefore, by increasing pH, the concentration of H^+^ ions decreases, which allows for better interaction between Pb^2+^ and the modified electrode surface, resulting in high detection currents at pH 5. In contrast, beyond this optimum, the current decreases because lead hydroxide precipitates at pH values over 5.5 (Dehaghi 2014). Therefore, pH 5 was selected as the optimum pH for further studies.

#### Effect of scan rate

**Fig. 7:**
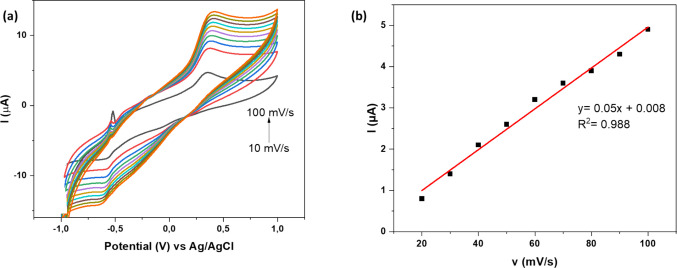
CV responses of (**a**) GCE/PTFE/Fe-alg-MOF at different scan rate (10–100 mv/s) in 1.0 M acetate-based electrolyte containing 1.0 mM Pb^2+^, (**b**) linear plot of current (µA) versus scan rate (mV s.^−1^)

As part of the analysis to understand the changes that occur with Pb^2+^ on Fe-alg-MOF/PTFE/GCE, CV studies were employed to show the entire redox process of Pb^2+^. The cyclic voltammetry of Fe-alg-MOF/PTFE/GCE in the presence of 1 mmol of Pb^2+^ was conducted at different scan rates (10–100 mV s^−1^) as shown in Fig. 7a. From the cyclic voltammogram, an oxidation peak of Pb^2+^ ion was observed at −0.6 V in all scan rates (10–100 mV s^−1^) (Fig. 7a). This indicated the conversion of Pb^2+^ to Pb^4+^ (Xu et al. 2024).

Furthermore, the relationship between the scan rate (10–100 mv/s) and peak current intensity was analyzed as shown in Fig. 7a. This was necessary as it assisted in establishing the electrochemical oxidation–reduction mechanism of electrocatalyst (Fe-alg-MOF) on Pb^2+^ ions. It was observed in Fig. 7b that the current peak intensity gradually increased with increasing scan rate, thus, showing a good linear relationship (*R*^2^ = 0.988) between scan rate and peak current. This linear relationship demonstrated that the oxidation reaction of Pb^2+^ was controlled by the adsorption process (Zhou et al. 2023). Furthermore, when the peak current was plotted against the corresponding square root of the scanning rate (Fig. S2a), a linear plot was attained (*R*^2^ = 0.996). This was regarded as an indication of a diffusion-controlled reaction in conjunction with an adsorption-controlled process, occurring at the modified electrode surface (Pu et al. 2023).

**Scheme 2:**
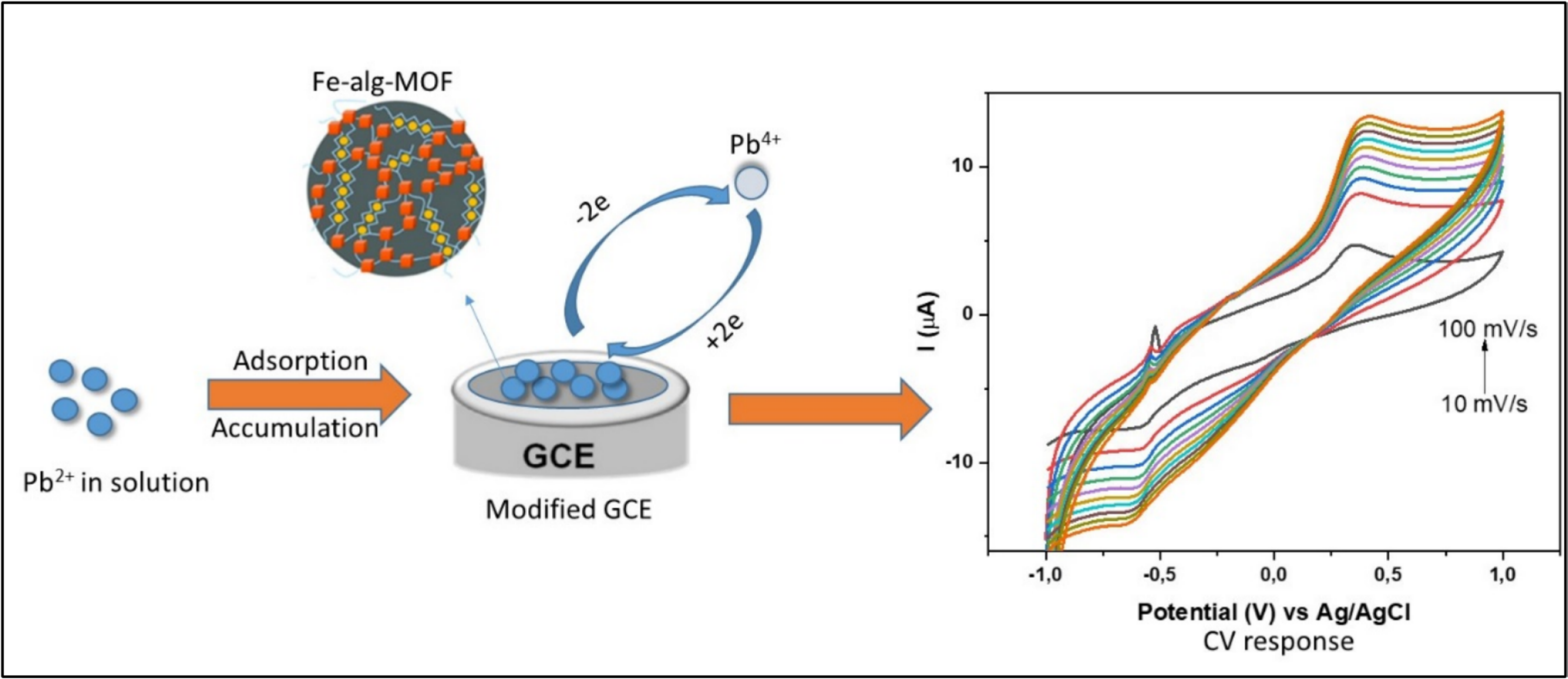
Possible mechanism on the detection of Pb^2+^ using Fe-alg-MOF-based sensor

Therefore, the prominent controlled process had to be identified by analyzing the relationship between logarithm of peak current and logarithm of scan rate. Results showed a good linear correlation (*R*^2^=0.992). Normally, a relationship between the scan rate (v) and current (I) is described by Eq. (3).

3 $$I=a{v}^{b}$$

where *a* and *b* are empirical parameters. When the value of b = 0.5, it indicates a diffusion-controlled process, and when a value of b = 1, it is an adsorption-controlled process (Zhou et al. 2023). The results (Figure S2b**)**, gave a slope of 0.90, indicating that the value of b was 0.90 which is closer to 1. These findings were similar to the results of a previous study (Zhou et al. 2023), confirming the adsorption-controlled process for Pb^2+^ on the surface of Fe-alg-MOF/PTFE/GCE. Therefore, the proposed mechanism in this study suggests that adsorption of Pb^2+^ ions on the modified electrode (Fe-alg-MOF) occurs first, followed by the diffusion of adsorbed ions onto the GCE surface as shown in Scheme 2.

#### Effect of Pb^2+^concentration

**Fig. 8:**
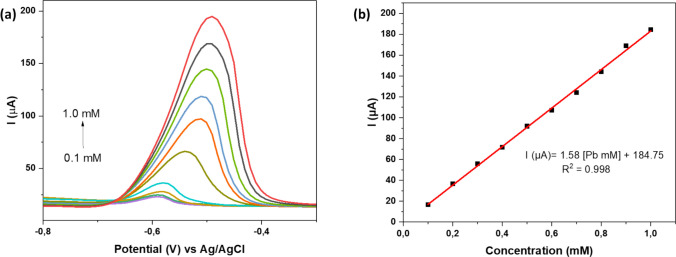
SWV obtained at Fe-alg-MOF PTFE/GCE for the (**a**) detection of Pb^2+^ at different concentration (0.1–1.0 mM) in 1.0 M ABS at 20 mV s^−1^ scan rate (**b**) Linear plot of Pb.^2+^ concentration (0.1–1.0 mM) vs current (µA)

Square wave voltammetry (SWV) was applied to investigate the analytical performance of Fe-alg-MOF/PTFE/GCE for the detection of Pb^2+^ ions. The electrochemical performance of the sensor towards different concentrations (0.1 to 1.0 mM) of Pb^2+^ was performed in ABS, using a scan rate of 20 mV. This was necessary to establish the analytical parameters such as the limit of detection (LOD) and limit of quantification (LOQ) which are related to the sensitivity of the analytical method. As seen in Fig. 8, the current was directly proportional to the concentration of Pb^2+^. This trend indicated that as the concentration increases, a greater number of Pb^2+^ ions reach the electrode surface, resulting in an increased current response (Zhou et al. 2023).

The LOD and LOQ values were calculated as 3 and 10 times the standard deviation to the slope (slope of the calibration curve) ratios (Lang et al. 2022), respectively. In this case, the standard deviations were computed from 5 blank samples. To determine the sensitivity of the electrochemical sensor, the regression equation in the lower concentration range was considered. The LOD and LOQ values for the proposed electrochemical sensor were 1 µM and 4 µM, respectively.

The analytical performance of Fe-alg-MOF/PTFE/GCE sensor was assessed and compared with previously reported MOF-based Pb^2+^ sensors (Table 4). As summarized in Table 4, several MOF-based sensors have achieved low detection limits, particularly hybrid systems incorporating additional functional components (Jiokeng et al. 2024; Saleem et al. 2025; Tan et al. 2023). However, high temperature synthesis, strong oxidizing agents and inert conditions are involved in the synthesis of most of these materials, limiting their scalability, cost, and environmental compatibility. In contrast, the Fe-alg-MOF/PTFE/GCE sensor exhibits a LOD of 1.0 µM with a linear range of 4–50 µM. The LOD of the developed sensor was comparable to previously reported MOF-based Pb^2+^ sensors (Lu et al. 2021; Wang et al. 2025; Xu et al. 2024; Ye et al. 2020) (Table 4). The sensing response was attributed to the presence of accessible Fe-based electroactive sites within the alginate-stabilized MOF network, which promoted Pb^2+^ adsorption and accumulation at the electrode surface. Furthermore, the Fe-alg-MOF/PTFE/GCE sensor showed better sensitivity compared to other sensors based on mesoporous N-rich carbon, polyvinylbutyral composite and reduced graphene oxide (Bhatia et al. 2025; Letsoalo et al. 2021; Mane and Joshi 2023). This performance was achieved without employing bimetallic frameworks, carbon nanomaterials, or biological recognition elements. Although the developed sensor does not achieve the lowest reported LOD, it offers a competitive balance of sensitivity, linear working range, material simplicity, and sustainability.

**Table 4:** Comparative analysis of different modified electrodes for Pb (II) detection

Material	Technique	LOD (µM)	Linear range (µM)	Reference
MOF-808-His	SWASV	0.01	0.001–0.5	Jiokeng et al. (2024)
NH_2_-UiO-66@ZIF-8//MWCNT	DPASV	0.001	0.003–70	Tan et al. (2023)
rGO-IIP	Chemoresistive	1.8	5–200	Letsoalo et al. (2021)
PAMAM/Ni-MOF	SWASV	1.2	1–100	Wang et al. (2025)
Bi-MOF/PANI	DPASV	0.1	0.025–20	Saleem et al. (2025)
Ln-MOF	SWASV	1.1 × 10^3^	-	Ye et al. (2020)
Ca-MOF	ASV	0.64	-	Pournara et al. (2019)
UiO-66	SWASV	1.1	10–50	Lu et al. (2021)
Mn(TPA)-MOF/SWCNT	DPASV	2.0	0.10 to 14.0	Cai et al. (2017)
PVB/PEDOT:PSS/MoS_2_	DPV	27.8	25–60	Mane and Joshi (2023)
ZIF-8@GO	DPASV	1.1		Y. Xu et al. (2024)
Mesoporous N-rich carbon	SWV	1.0	1–6000	Bhatia et al. (2025)
Fe-alg-MOF	SWV	1.0	4–50	This work

MOF-808-His: histidine-grafted metal–organic framework MOF-808; NH_2_-UiO-66@ZIF-8/CMWCNT: NH_2_-UiO-66@ Amino functionalized zirconium-based metal–organic framework@Zeolitic Imidazolate Framework-8/carboxylated multi-walled carbon nanotubes; rGO-IIP (reduced graphene oxide–ion-imprinted polymer); DPASV: Differential pulse anodic stripping voltammetry; PVB/PEDOT:PSS/MoS_2_: polyvinylbutyral and poly(3,4-ethylenedioxythiophene): poly(styrene sulfonate) modified with molebdenum disulphide; ASV: Anodic tripping voltammetry; Mn(TPA)-SWCNTs: manganese-terephthalic acid MOF/single-walled carbon nanotubes; PANI: polyaniline; PAMAM: Polyamide-amine dendrimers

### Repeatability, stability, reproducibility

**Fig. 9:**
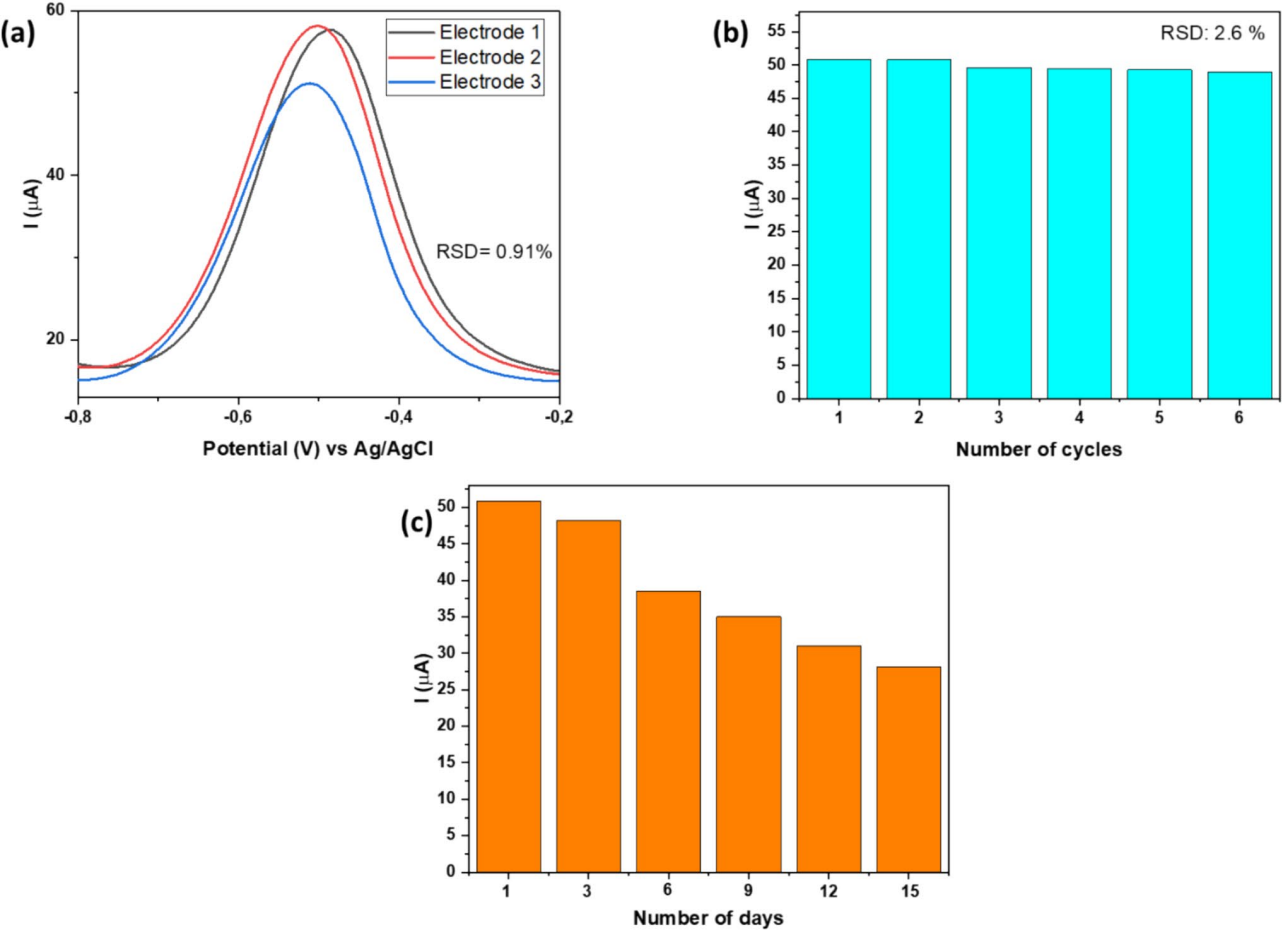
SWV response of 0.5 mM Pb^2+^ solution using Fe-alg-MOF/PTFE/GCE for (**a**) reproducibility studies, (**b**) repeatability studies (**c**) stability studies in 1.0 M ABS electrolyte solution at a scan rate of 20 mV s^−1^

The precision of the method was assessed through repeatability and reproducibility tests as shown in Fig. 9. The reproducibility of the Fe-alg-MOF/PTFE/GCE sensor was evaluated by detecting Pb2⁺ using three different electrodes, as illustrated in Fig. 9a. According to the reproducibility experiment, the Fe-alg-MOF/PTFE-modified sensor demonstrated appreciable reproducibility with a RSD of 0.9%. Furthermore, the repeatability of Fe-alg-MOF/PTFE/GCE was evaluated over six consecutive measurements under optimized conditions (Fig. 9b), yielding a RSD of 2.6%. This indicated a reproducible sensor as the RSD value was within an acceptable range.

The storage stability of Fe-alg-MOF/PTFE-based sensor was studied over a period of 15 days at different intervals, with the sensor being stored at 4 °C in the refrigerator when not in use. During the storage stability investigation, a slight decrease of less than 5% in current was observed after 3 days (Fig. 9c). This was comparable to the sensor reported by Yu et al. (2017), where the sensor lost only 7.6% of its initial electrochemical response over three days. However, as days progressed, the Fe-alg-MOF-based sensor demonstrated a gradual decline in performance, with nearly 50% of the initial current response lost by the 15th day. This reduction in signal was attributed to the possible swelling of alginate matrix. Comparatively, Alam et al. (2019) reported a multi-walled carbon nanotube/β-cyclodextrin/screen-printed electrode for the detection of Pb^2+^ that showed degradation after six days. Additionally, studies conducted by Tu et al. (2022), observed a similar decrease in performance with their alginate-based sensor for the detection of hydrogen peroxide. Even though the Fe-alg-MOF/PTFE sensor demonstrated promising short-term storage stability, its long-term performance was limited due to the inherent swelling behavior of the alginate matrix. Thus, there is a need for further structural optimization to enhance durability such as the incorporation of polyvinyl alcohol (PVA) to form an interpenetrating polymer network such as the work reported by Lee et al. (2021) and Mukundan et al. (2024). In addition, the incorporation of reinforcing fillers such as conductive material may assist in decreasing swelling of alginate (Hurtado et al. 2022; Musa et al. 2023).

### Interference studies

**Fig. 10:**
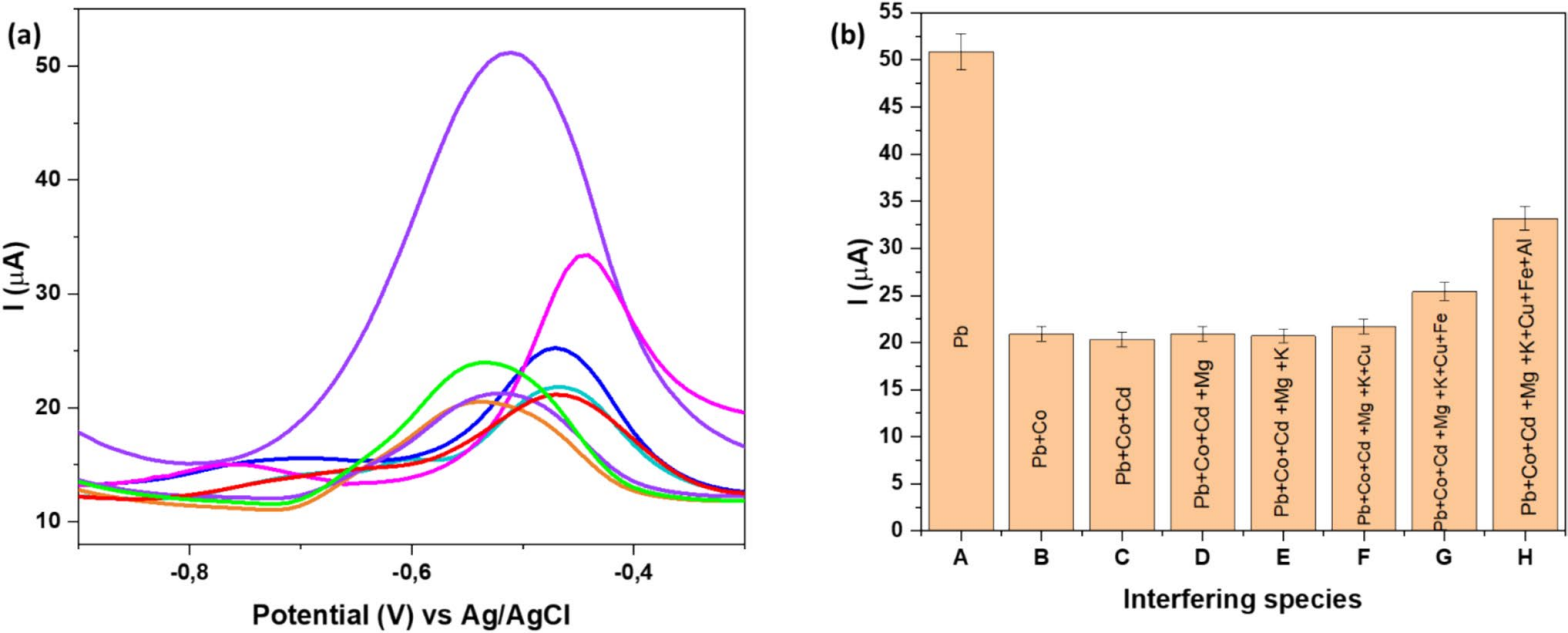
SWV response of (**a**) Pb^2+^ using GCE/PTFE/Fe-alg-MOF in the presence of different interfering species at a scan rate of 20 mV s^−1^ (**b**) Interference study in the presence of 0.5 mM Pb^2+^ and 1.0 mM of K^+^, Cu^2+^, Mg^2+^, Cd^2+^, Al^3+^, Co^2+^ and Fe^2+^ in 1.0 M ABS

Interference studies were performed to check the effect of certain interfering species like Cu^2^⁺, Mg^2^⁺, Cd^2^⁺, Al^3^⁺, Co^2^⁺, and Fe^2^⁺ in the presence of 0.5 mM in 1.0 M ABS on the fabricated Fe-alg-MOF/PTFE/GCE (Fig. 10). The selection of these interferent ions was influenced by their co-occurrence with Pb^2+^ in numerous environmental water samples (Fisher et al. 2021; Genthe et al. 2018; Madikizela et al. 2023). In this study, the concentration of Pb^2^⁺ was kept at 0.5 mM solution, while each competitor had a concentration of 1.0 mM. According to Fig. 10a, the Fe-alg-MOF/PTFE/GCE sensor showed a decrease in current Pb^2+^ when most interferents were introduced in the Pb^2+^ solution. Additionally, metal ions such as Fe^2+^, Al^3+^ and Cu^2+^ caused a potential shift to more positive potentials for Pb^2+^, indicating interference occurring between the analyte and interfering ions. The observed interference could be explained by the competition for active binding site on the surface of the modified Fe-alg-MOF/PTFE/GCE sensor, which is consistent with findings from other studies (Liu et al. 2022; Xiao et al. 2016). The developed sensor exhibited reduced selectivity to some metal ions (Fig. 10b), likely due to the adsorption process occurring on the modified electrode surface. These results show the potential of the Fe-alg-MOF/PTFE modifier to be used as a metal ion-adsorbent as it can interact with most metal ions under the optimized conditions. (Fig. 10a).

### Application to real sample

The practical application of the prepared GCE/PTFE/Fe-alg-MOF sensor was estimated by determining Pb^2+^ content in wastewater samples collected from Zwelitsha wastewater treatment plant in Eastern Cape, South Africa. Before analysis, the water samples were purified through filtration and the pH was adjusted to the optimum value (pH 5). The standard addition technique was applied for Pb^2+^ detection in all samples using SWV technique. Initially, the background SWV scan was tested from the collected sample. Next, known concentrations of Pb^2+^ were spiked into the water samples, and the resultant samples were analyzed using SWV. As shown in Table 5, the developed sensor was unable to detect Pb^2+^ in influent water samples possibly due to the complex matrix present in influent samples. However, in effluent water samples, recoveries ranging from 100.8 ± 4.2% to 101.2 ± 5.6% were obtained. It has been reported that iron-based sensors achieve excellent recoveries, sometimes exceeding 100%, which is reflective of a highly sensitive and accurate sensor (Jjagwe et al. 2024). Hence, the developed sensor demonstrated high accuracy in detecting Pb^2+^ in effluent water samples.

**Table 5:** Determination of Pb^2+^ in real wastewater, river and acid mine drainage samples

	Added (mM)	Found (mM)	Recoveries (%)	RSD (%)
Influent	0.4	0	-	-
	0.8	0	-	-
Effluent	0.4	0.3	100.8	4.2
	0.8	0.6	101.2	5.6

## Conclusion

This work demonstrates, for the first time, the use of a Fe-alginate-MOF synthesized in situ from synthetic AMD as a sustainable electrode material for Pb^2^⁺ electrochemical sensing. The Fe-alginate-MOF composite, incorporating PTFE as a binder, was employed under optimized conditions for the detection of Pb^2+^ in water samples. The electrochemical behavior of Fe-alginate-MOF/PTFE/GCE was characterized using EIS and CV, while Pb^2+^ detection was investigated using SWV technique. The developed sensor exhibited a linear response from 4 to 50 µM with a detection limit of 1.0 µM. Good reproducibility, robustness, and repeatability were obtained, with an average SWV peak current of 55.7 µA (RSD 0.9%) and a repeatability RSD of 2.6% over six consecutive cycles. Practical applicability was confirmed through analysis of real effluent samples, yielding satisfactory recoveries of 100.8 ± 4.2% to 101.2 ± 5.6%. The Fe-alginate-MOF/PTFE/GCE sensor was comparable with previously reported MOF-based sensors. However, signal decay over time suggests the need for further structural optimization to improve stability. Overall, the results highlight the potential of a waste-derived alginate-MOF composite as a practical electrochemical sensing platform for Pb^2+^ detection in waterbodies.

## Supplementary Information

Below is the link to the electronic supplementary material.

ESM 1 (DOCX 63.3 KB)

## Data Availability

Data will be made available on request.
