# Supplementary material for: Electrochemical sensor based on iron-based metal-organic framework nanocomposite derived from acid mine drainage for the detection of lead (II) ions in water
Source: Environ Sci Pollut Res Int. 2026 Mar 25;33(12):5621–39. doi: 10.1007/s11356-026-37670-7 (PMC13091878; doi:10.1007/s11356-026-37670-7)
Supplement: Supplementary file 1 — (DOCX 63.3 KB) [file 11356_2026_37670_MOESM1_ESM.docx]

**Figure S1:** ΔEp vs. square root of scan rate for Fe-alg-MOF electrode


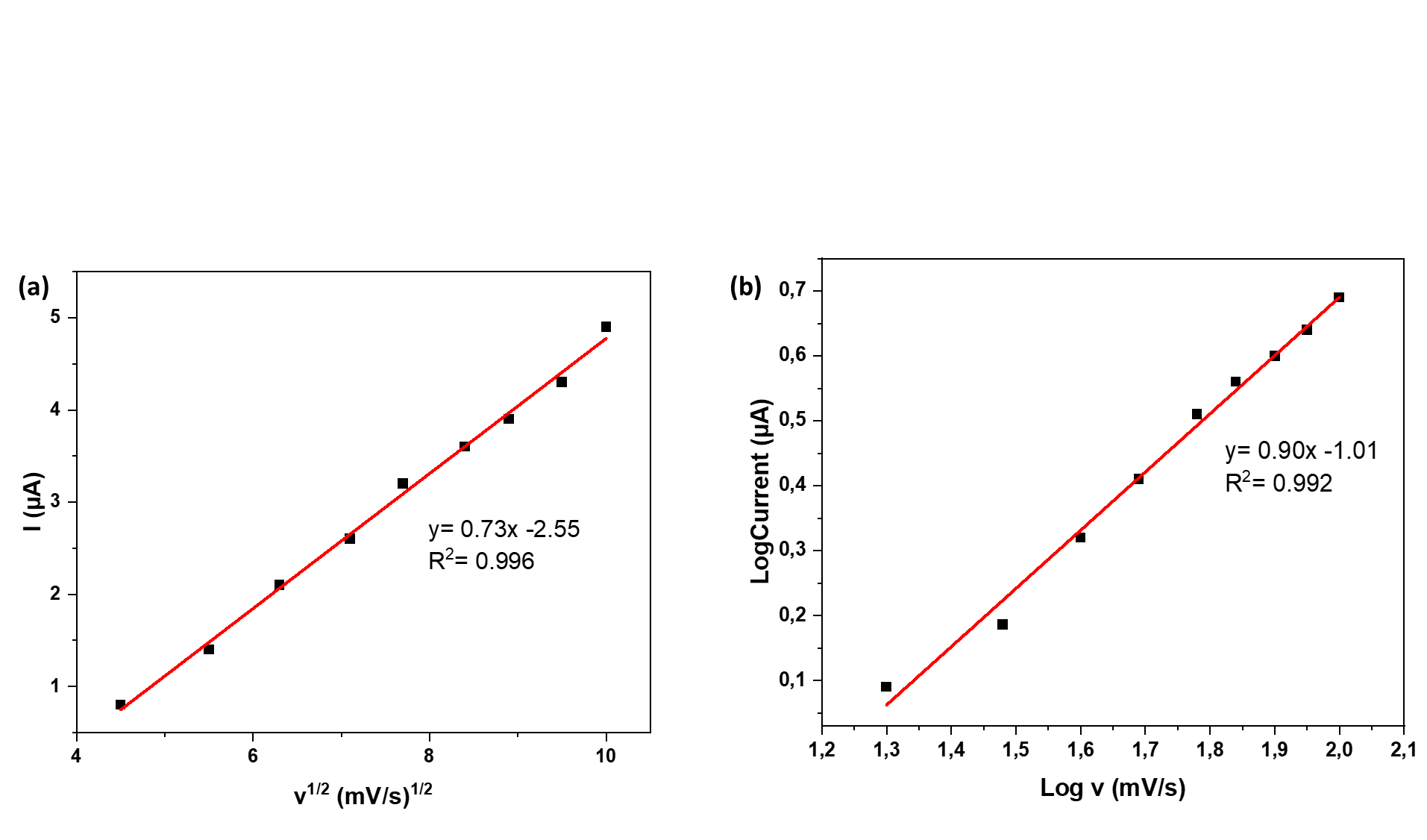


**Figure S2:** **(a)** linear plot of current versus square root of scan rate and **(b)** linear plot of log current versus log scan rate
